# Hemoglobin and Erythropoietin After Commercial Saturation Diving

**DOI:** 10.3389/fphys.2018.01176

**Published:** 2018-08-21

**Authors:** Fatima Z. Kiboub, Costantino Balestra, Øyvind Loennechen, Ingrid Eftedal

**Affiliations:** ^1^Department of Circulation and Medical Imaging, Faculty of Medicine and Health Sciences, NTNU: Norwegian University of Science and Technology, Trondheim, Norway; ^2^TechnipFMC, Stavanger, Norway; ^3^Environmental & Occupational Physiology Laboratory, Haute Ecole Bruxelles-Brabant (HE2B), Brussels, Belgium; ^4^Faculty of Nursing and Health Sciences, Nord University, Bodø, Norway

**Keywords:** hyperoxia, hypoxia, hematology, normobaric oxygen paradox, saturation diving

## Abstract

Saturation divers are exposed to elevated partial pressure of oxygen (ppO_2_) in their hyperbaric work environment. Experimental studies indicate that oxygen transport is altered, and we have previously reported a drop in hematocrit and extensive downregulation of genes involved in blood oxygen transport capacity after decompression from professional saturation diving. Here we investigate the initial period of hematological adjustment back to normobaric air after professional saturation diving. Erythropoietin (EPO) and hemoglobin (Hb) were measured in blood from 13 divers at two time-points after saturation assignments lasting up to 4 weeks; first immediately after decompression and again 24 h later. Pre-dive levels defined baselines. The ppO_2_ varied from 40 kPa in the saturation chambers during storage, 50 to 80 kPa during bell excursions, and gradually reduced to 21 kPa during decompression to surface pressure. EPO was similar to baseline immediately after saturation diving (*P* = 0.4), and markedly increased within the next 24 h (99%, *P* < 0.0002). Hb levels remained slightly reduced at both time-points (4% immediately after; *P* = 0.02, 8% 24 h after; *P* < 0.001). The results imply that the hematological acclimatization back to normobaric air was ongoing, but not completed, during the first 24 h after professional saturation diving.

## Introduction

Saturation diving is considered a safe method for human subsea interventions for extended periods of time. Commercial saturation divers live onboard dive support vessels in chambers compressed to a certain storage depth. They use pressurized diving bells to take them to the seabed/work location maximum 13 msw shallower or deeper than their storage depth; where they conduct different tasks from construction to maintenance. Several experimental studies into physiological acclimatization and possible harmful effects of saturation diving reported reduction in the production of reticulocytes, erythrocytes, serum erythropoietin, and hemoglobin; and cases of anemia (Nakabayashi et al., 1991; Thorsen et al., 2001; Hofso et al., 2005; Revelli et al., 2013). However, few investigations have been performed during real commercial saturation diving offshore. Indeed, the difference in conditions between dry and wet dives (Weathersby et al., 1990), including the use of hot-water suits (Hope et al., 2005) and the activity level during the dive; contribute to the stress levels faced during the dives (Møllerløkken et al., 2011). In a previous study of commercial saturation divers on the Norwegian Continental Shelf, the divers’ hematocrit levels were measured (Kiboub et al., 2018). Data showed they were reduced post-surfacing, a possible sign of physiological acclimatization to an elevated partial pressure of oxygen (ppO_2_) during saturation. Therefore, we decided to investigate the changes in hemoglobin (Hb) and erythropoietin (EPO) during the initial 24 h after decompression from a long commercial saturation dive, to see the divers’ readjustment when they move from a hyperoxic hyperbaric helium-oxygen (heliox) atmosphere to normobaric air. We hypothesized that the levels of Hb and EPO should be decreased immediately post-surfacing and start to increase during the first 24-h post-saturation with some delay for Hb increase after EPO increase.

## Materials and Methods

### Research Ethics

This study was organized from Norway, and performed on occupational saturation divers during work assignments in the United Kingdom, with TechnipFMC as a Diving Contractor. The study protocol was approved by the local Regional Committee for Medical and Health Research Ethics (REK), approval reference number 2015/351; and by TechnipFMC’s Diving and Health services in Norway and United Kingdom divisions. The data and sample collections were conducted according to the Declaration of Helsinki principles for ethical human experimentation. All participants gave informed, written consent before inclusion into the study, and held valid health certificates for working as saturation divers on the United Kingdom Continental Shelf. The study group consisted of males who were cleared for diving by the Diving Contractor’s hyperbaric nurse after the mandatory pre-dive medical examination. All were non-smokers, and without infections at the time of their pre-dive examination.

### Dive Procedure

The diving operations took place during September to November 2016 off the northeastern coast of Scotland, and were conducted according to the Diving Contractor’s procedures. The divers’ storage depths varied between 80 and 90 meters of seawater (msw), depending on the work location. A saturation period included compression, bottom time with bell runs, and decompression. During compression, the chamber was pressurized at 1 msw/min with 20 min stop at 10 msw for chamber leak checks before proceeding to the storage depth at which the divers were held. At storage depth, the divers had a 1 h stabilization period before bell runs commenced. Throughout the period at storage depth, the living chamber’s heliox atmosphere kept a ppO_2_ of 40 kPa. Each bell run lasts for up to 8 h with <6 h in-water per bell run. During bell diving, the ppO_2_ was maintained between 50 and 80 kPa. Between the third and fourth hour of in-water dive time, the divers had to return to the diving bell for a 20-min restitution break for rehydration. The work tasks conducted on the seabed included, but were not limited to, pipelines tie-ins, handling of rigging and equipment and installation of flexible jumpers and umbilical. During decompression, a ppO_2_ of 50 kPa was kept up to 12.7 msw; then the ppO_2_ was gradually decreased while the oxygen content in the heliox was kept between 21 and 23% until surfacing. The ascent rate was 1.5 msw/h until arriving to 15 msw; from thence to surface, the ascent rate was 0.6 msw/h. The decompression was stopped for 5 h after every 19 h of ascent, for the divers to stabilize.

Four divers stayed in saturation for 25 days (d) and 15 h, *n* = 5 stayed 27 days and 6 h and *n* = 4 stayed 27 days and 21 h. The total duration of saturation depended on the operations; with a maximum of 28 days as per the Diving Contractor’s requirements. After surfacing, the divers stayed on the vessel for at least 12 h for “bends watch.” They were instructed to rest and avoid physical exertion during the first 24 h.

### Blood Sampling

Blood samples were collected by standard antecubital venipuncture; in VACUETTE^®^ Z Serum Sep Clot Activator 5 ml gel tubes (Greiner Bio-One, Radnor, PA, United States), prior to saturation during the pre-dive medical examinations; at the end of decompression during the post-dive medical examinations, always within 2 h before or after saturation diving. The final sample was collected 24 h after the end of the decompression. Sampling was conducted between 08:00 and 24:00 h, depending on the time of day when the divers emerged from the chamber.

Hb was measured immediately after blood collection. A capillary pipette was used to transfer a 10 μl drop of blood from the tube and deposit it into the well of a disposable test strip attached to a portable Mission Plus Hb apparatus (ACON Laboratories, San Diego, CA, United States). The same apparatus was used for all measurements. Control strips were used regularly to check analytic performance. The manufacturer indicated that the apparatus’s accuracy was ±0.4 g/dl for Hb. All Hb values were within the reported normal range for healthy males (14–18 g/dl) (Billett, 1990). The within-subject variation coefficient for Hb was 0.44 g/dl (Lacher et al., 2012).

Within 30 min of sampling, the blood tubes were centrifuged in an EBA 270 centrifuge (Hettich GmbH & Co.KG, Tuttlingen, Germany) at 4,000 RPM (1,800 G) for 10 min at room temperature to separate the serum. The serum was stored frozen at -20°C onboard the vessel before being transported in cooling cubes (VeriCor Medical Systems, Holmen, WI, United States) at 4°C to NTNU, where it was kept frozen at -80°C until analysis. Transportation from the vessel to the analytic facility took 12–18 h.

EPO measurements were made as a single-batch run on a Siemens DPC IMMULITE 2000 Immunoassay System (Siemens Healthcare GmbH, Germany) in the IEC 17025-accredited laboratory at the St Olav’s University Hospital, Department of Clinical Chemistry, Trondheim, Norway. The laboratory reported analytical variation of 8.6%. With one exception, (EPO 37.7 IU/l), all EPO values were within the normal range, 4.3–29.0 IU/l, reported by the laboratory.

### Statistical Analysis

Prior to the analyses, the data were assessed for normality using the Shapiro-Wilk test. Hb data was normally distributed, whereas the EPO data became so after log transformation. Grubbs’ test was used to examine the data for outliers: none where identified. Percentages changes relative to pre-saturation diving values were calculated for each parameter at 0 and 24 h post-saturation diving. A repeated measures ANOVA with a Dunnet’s *post hoc* test was performed. Paired *t*-tests were conducted for group-wise comparison of data 0 h post-dive to 24 h post-dive. All statistical tests were done using a standard computer package, GraphPad Prism version 7.00 (GraphPad Software, San Diego, CA, United States). *P*-values < 0.05 were considered significant.

## Results

The dives were completed in accordance with the Diving Contractor’s saturation diving procedures, without adverse events. Out of 20 divers originally enrolled in the study, seven were excluded as they left the vessel shortly after surfacing, before the 24 h post-dive blood samples could be obtained. One diver had a bacterial infection on his face during decompression; he was given antibiotics but not excluded from the study. See **Table 1** for a summary of the study participants’ demographics, diving history, body-mass index (BMI) and resting heart rate pre- and post-saturation diving.

**Table 1:** Description of study group.

				Pre-dive		Post-dive	
	Age (years)	Total diving (years)	Saturation diving (years)	BMI (kg/m)	Heart rate at rest (beat/min)	BMI (kg/m)	Heart rate at rest (beat/min)
**Mean (range)**	45.8 (34.0-59.0)	21.4 (6.0-35.0)	15.8 (3.0-34.0)	26.6 (23.3-31.1)	67.3 (54.0-91.0)	26.4 (22.2-31.4)	75.4 (58.0-96.0)


*n = 13, mean (range) pre- and post-dive.*

**FIGURE 1:**
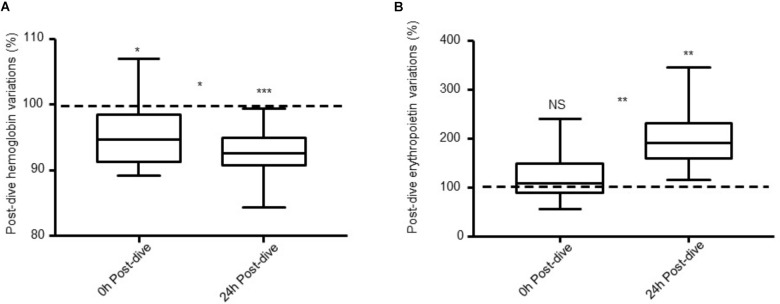
**(A)** Hb and **(B)** EPO levels 0 h post-dive and 24 h post-dive expressed as percentages of pre-saturation diving levels (dotted line) in 13 saturation divers. Error bars are ± SE. ^∗^*P* ≤ 0.01; ^∗∗^*P* ≤ 0.001; ^∗∗∗^*P* ≤ 0.0001 and NS, non-significant.

Hb and EPO data are shown in **Figure 1**. Pre-saturation diving values were used as a baseline for each diver to avoid the intra-subject variance. Hb fell during the dive, and continued to do so during the initial 24 h after saturation diving. Immediately after decompression, Hb was reduced by 4% relative to the pre-dive values (*P* = 0.01); and 24 h post-dive the Hb was reduced by 8% (*P* < 0.0001).

EPO was unchanged immediately after decompression relative to the pre-dive values (*P* = 0.4). However, during the 24 h post-dive, there was a marked increase in which EPO levels almost doubled (99%, *P* = 0.0002). Between 0 and 24 h post-saturation dive, there was a decrease in Hb levels (*P* = 0.02) and an increase in EPO (*P* < 0.001).

## Discussion

In this study, Hb levels were reduced after decompression from commercial saturation diving, and remained below their pre-dive levels 24 h later. The observed reduction of Hb immediately after decompression is in agreement with findings in a recent report (Deb et al., 2017). Whereas they found Hb no longer to be significantly reduced 24 h later, this present study observed a further decline. However, the number of subjects in both studies was limited, and our results are not necessarily conflicting. In a previous study of saturation divers, bilirubin levels were unchanged post-dive; indicating that the erythrocytes were not hemolyzed but rather their production was reduced, in line with the reduction of Hb production (Hofso et al., 2005). It is a challenge to maintain the divers’ hydration levels during bell excursions in commercial saturation diving. In this study, measurements of hematological status were done after completion of the saturation diving assignment, and not after the bell runs. After the last bell run, it took more than 5 days to complete the decompression, during which time the divers were encouraged to keep their hydration levels high in order to facilitate efficient gas exchange as dehydration is known to interfere with plasmatic surface tension and increased bubble production during decompression (Gempp et al., 2009).

The EPO levels in this study were unchanged immediately post-saturation diving, but markedly increased during the next 24 h. During the long saturation period, the divers acclimatize to a ppO_2_ of 40 kPa, which is close to twice the ppO_2_ in normal air (Revelli et al., 2013). EPO down-regulation leads to a decrease in the production of erythrocytes (Krantz and Jacobson, 1970), working to reduce the toxicity caused by the increased concentration of reactive oxygen species (ROS) at high ppO_2_. EPO is up-regulated when a reduction is sensed in the breathing gas oxygen content; which occurs after the divers emerge from the hyperbaric chamber and readjust to life in ambient air (Nakabayashi et al., 1991). Moving from hyperbaric hyperoxia to normobaric normoxia during the first 24 h after decompression causes a relative hypoxia. During this phase, up-regulation of EPO triggers erythrocyte production. This phenomenon, known as the normobaric oxygen paradox (NOP), was first described in healthy subjects breathing normobaric oxygen (Balestra et al., 2004); and later reported in experimental deep saturation dives (Hofso et al., 2005). The primary endogenous antioxidant glutathione (GSH) scavenges ROS in hyperoxic hyperbaric environments by oxidation. When GSH is oxidized, it forms glutathione disulphide (GSSH) which takes time to return to its reduced state. The reduction process is catalyzed by the enzyme GSH reductase using NADPH, the efficiency of which depends on the glucose conversion rate. The slow rate of the latter leads to GSH depletion and accumulation of GSSH, which inhibits the activation of the transcription factor hypoxia inducible factor-1 alpha (HIF-1α) via the hypoxia-responsive element (HRE) (Cimino et al., 2012). Binding of HIF to HRE controls the expression of genes involved in oxygen homeostasis, including EPO (Semenza et al., 1996; Masson et al., 2001). Under stable normoxic conditions, HIF-1α is inactivated by binding to Von Hippel Lindau (VHL) protein; this complex is constantly bound to ubiquitin ligase (Maxwell et al., 1999). But when returning to normobaric normoxia or in the case of our divers to a relative hypoxia; the GSH ratio increases. The extra GSH will neutralize ROS and inhibit the binding of HIF-1α to VHL; which leads to the activation of HIF-1-mediated gene transcription. This triggers the production of EPO; thus, increasing the hemoglobin and erythrocytes production (Huang et al., 1997; De Bels et al., 2012).

In a previous experiment (Revelli et al., 2013), six divers were kept in a habitat at 9 msw; breathing air at 40.5 kPa ppO_2_ for 14 days. Before the experiment, EPO was 11.6 ± 3.1 IU/l. This decreased by the 14th day of saturation to 4.2 ± 1.6 IU/l, and at the end of the experiment, EPO was 4.5 ± 1.7 IU/l. At this point, one of the divers displayed mild signs of anemia. The authors discussed that the prolonged hyperoxia might have caused a clinically relevant anemia if the exposure was prolonged. In the present study’s setup, despite the higher ppO_2_ and longer exposure times, the results show a different trend. EPO levels trended toward a decrease at the end of the decompression before increasing, but still – with one exception – they remained within the normal range. To our knowledge, a transient increase in EPO is not associated with disease risk in healthy individuals. Also, even though total duration of the diver’s stays in saturation differed, this did not affect the outcome.

### Limitations

Hematological variables may be subject to circadian and other temporal variations. Studies of circadian variation in EPO have come to different conclusions (Pasqualetti and Casale, 1996; Roberts and Smith, 1996; Balestra et al., 2004; Gunga et al., 2007) and it was not determined whether circadian rhythms had an impact in the present study. Due to operational restraints, it was not possible to measure the hydration state of the divers throughout the saturation period, nor to control the timing of blood sampling as this depended on the divers’ availability on-board. Therefore, variations associated with circadian rhythms or dehydration could not be ruled out.

## Conclusion

Blood Hb was reduced immediately after decompression from commercial saturation diving, compatible with acclimatization to the hyperbaric hyperoxia the divers experienced during saturation. A marked increase of EPO levels 24 h after decompression suggested re-acclimatization back to breathing normobaric air, advocating for a physiologically compensated effect of the dive procedure.

## Author Contributions

FK, IE, and CB designed the study. ØL obtained the necessary consents. FK managed the blood and data sampling. CB conducted the statistical analysis. FK initiated the manuscript and all the co-authors contributed to the analysis, writing and approval of the final manuscript.

## Conflict of Interest Statement

FK and ØL were employed by TechnipFMC in Norway. The remaining authors declare that the research was conducted in the absence of any commercial or financial relationships that could be construed as a potential conflict of interest. The reviewer NN and handling Editor declared their shared affiliation at the time of the review.
